# Discharge interventions for First Nations people with a chronic condition or injury: a systematic review

**DOI:** 10.1186/s12913-023-09567-5

**Published:** 2023-06-09

**Authors:** Julieann Coombes, Andrew J.A Holland, Courtney Ryder, Summer May Finlay, Kate Hunter, Keziah Bennett-Brook, Phillip Orcher, Michele Scarcella, Karl Briscoe, Dale Forbes, Madeleine Jacques, Deborah Maze, Bobby Porykali, Elizabeth Bourke, Camila A. Kairuz Santos

**Affiliations:** 1 The George Institute for Global Health, Level 5/1 King Street, Newtown, NSW 2042 Australia; 2 Department of Paediatric Surgery, The Children’s Hospital at Westmead, Corner Hawkesbury Road and, Hainsworth St, Westmead, NSW 2145 Australia; 3 Indigenous Health College of Medicine and Public Health, Flinders University, Adelaide, SA 5042 Australia; 4 School of Health and Society, Wollongong University, Wollongong, NSW 2522 Australia; 5 Agency for Clinical Innovations, 1 Reserve Rd, St Leonards, NSW 2065 Australia; 6 The Sydney Children’s Hospital Network (SCHN), Sydney, NSW 2145 Australia; 7 National Association of Aboriginal and Torres Strait Islander Health Workers and Practitioners (NAATSIHWP), 31-37 Townshend Street, Phillip ACT, 2606 Australia; 8 Department Community and Justice NSW, Sydney, NSW 2012 Australia

**Keywords:** Aboriginal, Chronic conditions, Discharge Planning, First Nations, Injury, Torres Strait Islander

## Abstract

**Background:**

Aboriginal and Torres Strait Islander peoples have a unique place in Australia as the original inhabitants of the land. Similar to other First Nations people globally, they experience a disproportionate burden of injury and chronic health conditions. Discharge planning ensures ongoing care to avoid complications and achieve better health outcomes. Analysing discharge interventions that have been implemented and evaluated globally for First Nations people with an injury or chronic conditions can inform the implementation of strategies to ensure optimal ongoing care for Aboriginal and Torres Strait Islander people.

**Methods:**

A systematic review was conducted to analyse discharge interventions conducted globally among First Nations people who sustained an injury or suffered from a chronic condition. We included documents published in English between January 2010 and July 2022. We followed the reporting guidelines and criteria set in Preferred Reporting Items for Systematic Review (PRISMA). Two independent reviewers screened the articles and extracted data from eligible papers. A quality appraisal of the studies was conducted using the Mixed Methods Appraisal Tool and the CONSIDER statement.

**Results:**

Four quantitative and one qualitative study out of 4504 records met inclusion criteria. Three studies used interventions involving trained health professionals coordinating follow-up appointments, linkage with community care services and patient training. One study used 48-hour post discharge telephone follow-up and the other text messages with prompts to attend check-ups. The studies that included health professional coordination of follow-up, linkage with community care and patient education resulted in decreased readmissions, emergency presentations, hospital length of stay and unattended appointments.

**Conclusion:**

Further research on the field is needed to inform the design and delivery of effective programs to ensure quality health aftercare for First Nations people. We observed that discharge interventions in line with the principal domains of First Nations models of care including First Nations health workforce, accessible health services, holistic care, and self-determination were associated with better health outcomes.

**Registration:**

This study was prospectively registered in PROSPERO (ID CRD42021254718).

**Supplementary Information:**

The online version contains supplementary material available at 10.1186/s12913-023-09567-5.

## Background

Patients with severe injury or chronic health conditions often require long-term management by a multidisciplinary team of health experts [1]. Such management requires coordinated care between tertiary and primary community healthcare services and ongoing care to avoid complications and achieve better health outcomes [2, 3]. Research on strategies to improve chronic disease care is extensive and there is currently strong evidence supporting the use of community resources, telehealth, self-management and discharge planning to improve patient outcomes [4].

Discharge planning is paramount to improve the care transition from hospital to home by ensuring continuity of care [5, 6]. Appropriate discharge planning results in positive outcomes including reduction in the hospitalisation Length Of Stay (LOS), decreased unplanned readmission, and higher patient satisfaction [6–10]. To achieve better health outcomes, discharge planning should start prior or on admission to the hospital rather than after discharge, it should be patient centred, individualised and promote patient empowerment [6].

Within this manuscript we refer to indigenous people from all countries as “First Nations people” and use the words “Aboriginal and Torres Strait Islander” to refer specifically to Indigenous people of Australia”. First Nations peoples in colonised countries such as Australia, Canada, New Zealand and multiple countries in South America and Europe are culturally diverse and unique. They all, however, share a history of genocide, trauma, dispossession and discrimination that continues to have a negative impact on their health and wellbeing [11]. First Nations people globally have poorer health outcomes compared to non-Indigenous people in the same country and experience higher disease burden and mortality [11, 12]. For example, in New Zealand, Canada and the United States, First Nations people have a life expectancy of between 5 and 8.5 years lower than their non-indigenous counterparts [12, 13]. In Australia, Aboriginal and Torres Strait Islander people born between 2015 and 2017 had 8.6 and 7.8 fewer years of life expectancy compared to male and female non-Indigenous Australians, respectively [14]. In addition, with global increases in urbanisation of traditional lands, a disproportionate burden of chronic health conditions among First Nations people is worsening [15, 16].

Health inequities for First Nations people are shaped by colonisation and continue to be reinforced by current healthcare systems. Current models of care, including discharge planning services, have been developed using Western biomedical frameworks of health that do not align with First Nations people’s holistic definitions and concepts of health [17]. In addition, healthcare systems degrade First Nations’ worldviews, display systemic racism and fail to meet First Nations peoples’ needs [18–23]. Due to their diversity, First Nations communities around the world and within countries hold unique knowledge systems and health paradigms [24]. However, unlike the Western biomedical model, they have in common a concept of health that includes strong emotional and spiritual wellbeing and extends beyond the individual person to include the family, community and environmental wellness [24, 25]. The misalignment between Western biomedical health service delivery and First Nations peoples’ needs, and the racism and oppression exerted by the former, reinforces marginalisation contributing to the First Nations health gap [15]. Ongoing health disparities experienced by First Nations people and increasing recognition of culturally unsafe healthcare systems have driven a call for the global development and delivery of culturally appropriate and safe healthcare services for First Nations peoples [26–28].

In Australia there are two different groups of First Nations people, Aboriginal and Torres Strait Islanders: each group are distinct in their own cultural protocols [29]. Within these two groups, there is diversity with over 500 First Nations across Australia [30]. Aboriginal and Torres Strait Islander peoples have inhabited Australia since time immemorial, with connections to country, family and community remaining strong, despite colonisation and its impact on individuals and communities’ health and wellbeing. For example, the hospitalisation rate for Aboriginal and Torres strait Islander people remains 2.3 times the rate of non-Indigenous Australians and have higher relative risk of unplanned readmission or death after hospital discharge compared to non-Indigenous Australians [31–35]. Overall, Aboriginal and Torres Strait Islander people experience 284 years lost per 1000 people due to premature death and disease and it has been estimated that chronic disease including injury cause more than half (64%) of this disease burden [32]. Despite strong evidence on the benefits of ensuring ongoing care after hospital discharge through discharge planning, Aboriginal and Torres Strait Islander people with chronic conditions continue to face barriers to aftercare such as lack of communication, long distances to medical treatment, racism and culturally unsafe services [36–39]. These barriers have been identified through research that listened to the voices of community members and has informed this review.

Aboriginal and Torres Strait Islander people and other First Nations people have similar experiences with healthcare services and thus, there has been a global call for the implementation of services that are culturally safe for First Nation peoples. For this reason, we conducted a systematic review to gather evidence on discharge interventions that have been implemented worldwide to optimise after-care care for First Nations peoples with injury or chronic conditions. This review will inform the design and implementation of strategies to ensure Aboriginal, and Torres Strait Islander people with injury and chronic conditions have ongoing access to culturally safe care. The aims of this systematic review are to apply a decolonised approach:

1. To identify what discharge interventions have been implemented and evaluated globally for First Nations people with an injury or a chronic condition.
2. To identify what effect on health outcomes have been found with the interventions applied.

## Methods

The protocol for this systematic review has been registered in PROSPERO (ID CRD42021254718) and published [40]. We followed the reporting guidelines and criteria set in Preferred Reporting Items for Systematic Review (PRISMA 2020)[41]. A PRISMA checklist demonstrating the recommended items to include in a systematic review protocol and the location of each item in the document can be found in **Additional file 1.**

All authors of this paper acknowledge the harmful impact that Western research methods continue to have on First Nations peoples. The conduct of health research on First Nations people has historically been conducted by non-Indigenous researchers who have a colonial frame of reference, and overall standpoint which focusses on Western worldviews, epistemologies, ontologies and axiology [42]. These research frameworks have informed current policy and models of care [43, 44]. Far from achieving a positive impact for First Nations people, this way of conducting research has only served to reinforce inequities and systemic racism by portraying First Nations people as problematic others unable to achieve positive outcomes and who need “to be fixed” [42, 43].

Within this systematic review we used a decolonising approach in which Aboriginal and Torres Strait Islander worldviews and voices were prioritised and power was shifted from dominant Western-centred values and beliefs to Aboriginal and Torres Strait Islander ways of knowing, being and doing [42, 45]. This was done in three ways. First, engaging a research team lead by an Aboriginal Senior researcher (JC) and comprised mainly by Aboriginal and Torres Strait Islander people (CR, SMF, KBB, PO, MS, KB, DF, BP, EB). Aboriginal and Torres Strait Islander authors ensured that conclusions were drawn privileging Aboriginal and Torres Strait Islander cultural worldviews and beliefs. Second, we applied the consolidated criteria for strengthening the reporting of health research involving Indigenous Peoples (CONSIDER) statement to assess the reporting of health research involving Indigenous Peoples. Third, this study had Aboriginal and Torres Strait Islander Governance through an Aboriginal Reference Group comprised of Aboriginal and Torres Strait Islander researchers and community members.

### Search strategy

A systematic search was conducted for published and grey literature using the following databases: PubMed, CINAHL, ProQuest, Embase, Web of science, Google, and Google scholar. To locate grey literature, we reviewed the first 10 pages of Google results. We developed a search strategy using the Boolean operators “AND” and “OR” and a combination of key terms related to “Indigenous”, injury”, “chronic conditions” and “discharge intervention”. The search terms were adapted to each database. The search strategy used in each database is available in **Additional file 2.** Reference lists of the included studies were also checked for any additional articles that might meet inclusion criteria.

### Data collection and analysis

All search results were exported to the data manager EndNote X9. After removing duplicates, one Aboriginal and another non-Indigenous reviewer (JC, CK) independently screened titles and abstracts to select eligible papers. Pre-selected studies were then full text assessed according to the inclusion criteria (Table 1).

**Table 1:** Inclusion and Exclusion criteria for Review

Inclusion	Exclusion
1. Studies published in English between 01/01/2010 and 01/07/2022. 2. Studies including First Nation people with an injury or chronic condition. 3. All age groups. 4. Studies conducted in any country. 5. Evaluated the implementation of a discharge plan or any discharge intervention in any healthcare setting. 6. Primary studies using mixed methods or qualitative or quantitative methods [case-control, cross-sectional, cohort, randomised controlled trials (RCTs) and controlled clinical trials (CCTs)]	1. Studies conducted on non-Indigenous people. 2. Published in a language different than English. 3. Evaluated an intervention different than a discharge plan or related to patient discharge. 4. Didn’t evaluate any discharge plan or discharge related intervention. 5. Studies found only as abstract, letters to the editor, editorials, and reviews.

Data was extracted by CK and organised in an Excel (Microsoft, Redmond, CA) spreadsheet: study information (title, year of publication, country, authors, type of document, and journal) population and setting (sample size, sample source, age range, health condition, ethnicity, health setting) study methods (objectives, study design, quantitative or qualitative methods used, research tool and evaluated outcomes) and results (resulting themes, and associations). Author JC reviewed all extracted data to check for errors.

A descriptive synthesis was conducted independently by reviewers JC and CK to analyse included studies. Discrepancies between the reviewers at any stage of the data collection and analysis was resolved through discussion until consensus was reached. As part of our decolonising approach, all Aboriginal and Torres Strait Islander authors reviewed the data analysis and provided feedback on the interpretation of findings based on their own cultural and decolonising research expertise. This ensured that data analysis and conclusions reflect Aboriginal and Torres Strait Islander beliefs and health paradigms.

### Quality assessment

Included studies were assessed on their quality using the Mixed Methods Appraisal Tool (MMAT) [46]. The MMAT has previously been shown to be a comprehensive tool for assessing mixed method studies and meets the accepted standards for validity and reliability [47, 48]. Two independent reviewers assessed the quality of each study. Studies that scored “yes” to 0–1 items were classified as low quality, studies that scored “yes” to 2–3 items were classified as medium and those studies scoring “yes” in 4–5 items were classified as high quality. Additionally, JC and CK assessed the extent to which included studies adhered to the criteria listed in the consolidated criteria for strengthening the reporting of health research involving Indigenous Peoples (CONSIDER) statement. This was designed to strengthen the reporting of health research involving First Nations peoples and promote research approaches that are underpinned by First Nations participation, knowledge, and priorities to advance Indigenous health outcomes [49]. For the purpose of this review a “Yes” response was given when there was explicit and clear information indicating that the checklist item was addresses during research design or conduct. A “partially” response was given if the article had some information and a “No” response when there was no information indicating whether or not the item was addressed.

## Results

**Fig. 1:**
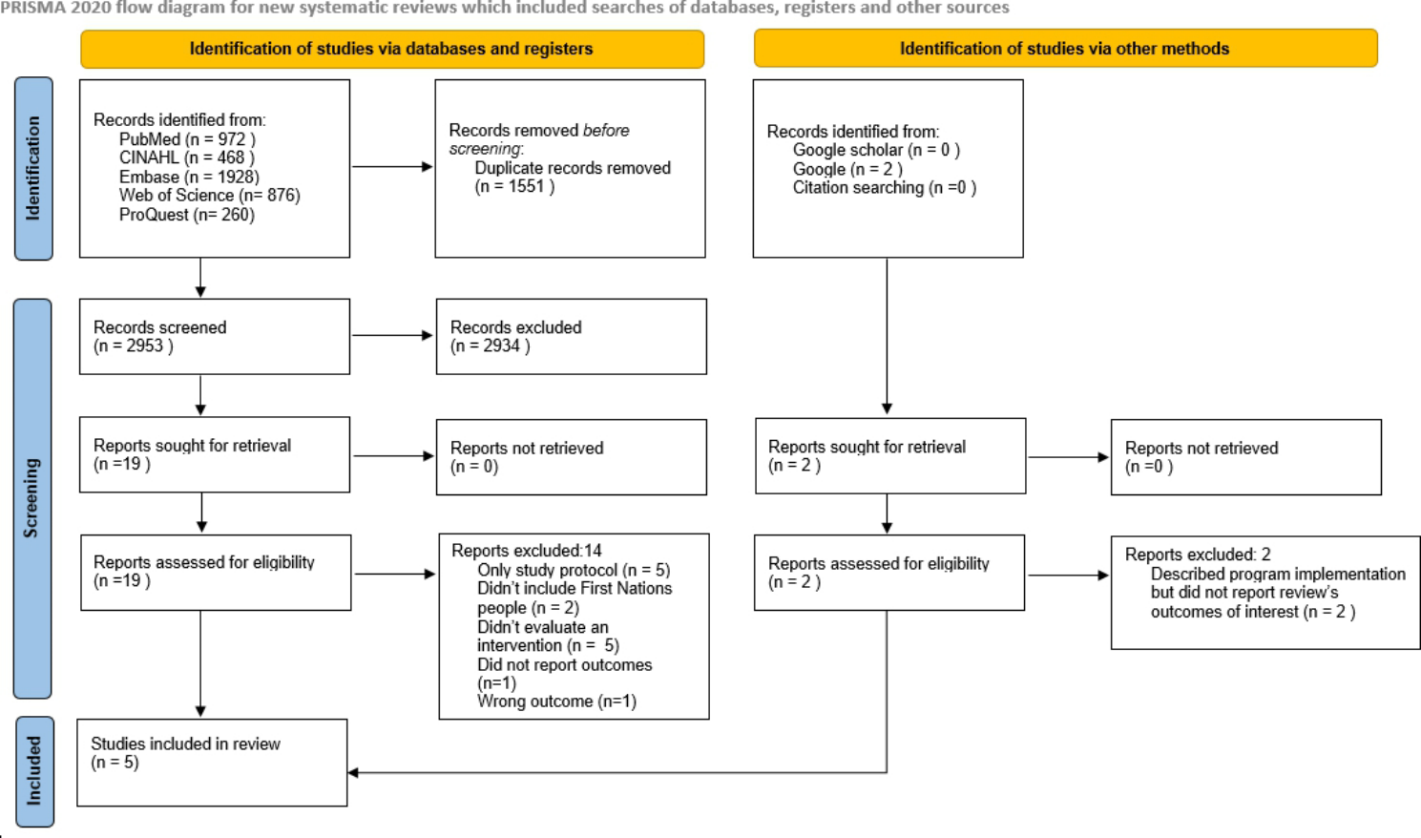
PRISMA flow diagram

The database search identified 4504 records and another 2 records were identified through manual search using Google. After removing 1551 duplicates, 2953 titles and abstracts were screened against inclusion criteria. Nineteen studies were full text assessed and five finally included in this systematic review. The screening process is depicted in a PRISMA flow diagram [41] **(**Fig. 1.**)**.

The majority (n = 4) of studies were conducted in Australia, more specifically New South Wales [50, 51], Northern Territory [52] and Western Australia [53] with the other study conducted in Hawaii [54]. The oldest study was published in 2014 [7] and the most recent one in 2021 [51]. Two studies focussed on children younger than 16 years [52, 53], two were conducted with people over 15 years old [50, 54] and one did not specify the age group [51]. From the five included studies, four were quantitative [50, 52–54] and one was qualitative [51]. Of the quantitative studies, one was a randomised controlled trial [52], two were before-and-after time series studies [53, 54] and the remaining study was a retrospective cohort [50]. Sample sizes ranged from 49 [51] to 18,659 participants [50]. The characteristics of the studies are summarized in **Additional file 3.** Three focussed on participants with a variety of chronic conditions including diabetes, cardiovascular disease, pulmonary and renal disease [50, 51, 54], one included any child who needed hospitalisation or out of hospital services [53] and the other (n = 1) included children with acute or chronic tympanic perforation [52].

### Interventions

Three studies conducted interventions using cultural experts (Aboriginal liaison officer or First Nations community health worker) to provide patient support and coordinate follow-up medical appointments [51, 53, 54]. The intervention of these three studies included linking the participants with community resources such as follow-up appointments close to home and other social services [51, 53, 54]. Other interventions included patient education and training in ambulatory care [51, 53], healthy lifestyle changes and medication use [54]. One study used a 48 h post-discharge telephone follow-up [50] and another used seven (one every 4 days) Multimedia Messaging Service (MMS) with short videos of First Nations role models (i.e. Elders or grandparents) who spoke in local language, surrounding the importance of hearing in an First Nations context, with a prompt to visit the clinic for the health check-ups [52].

### Outcomes

Hospital readmission was analysed by three studies [50, 51, 54]. Three studies analysed unplanned Emergency Department (ED) presentations [50, 51, 53]. Patient satisfaction was analysed in two studies [51, 52]. Other outcomes examined by individual studies included: hospital LOS [53], appointment non-attendance [53], post-discharge mortality within 28 days [50], number of clinic appointments attended [52], ear health after intervention [52] and hospital staff members’ and community service providers’ views on continuity and coordination of patient care [51].

Cresp et al. (2016) found that partnerships with primary care providers in the community, nurse-led coordination for follow-up appointments, education and training for ambulatory care were associated with a decrease in ED presentations, hospital admissions, non-attended appointments and hospital LOS [53]. Kim et al. (2019) also found a reduction in 30 days readmission rates when assigning a First Nations community health worker to coordinate follow-up appointments, linking to community resources and providing patient education on healthy lifestyle and medication use [54]. Similarly, Blignault et al. (2021) reported a decrease in hospital readmissions and ED presentations and improved patient experience with a model that included a transfer of care planning by a team of healthcare community service providers and patient and family education regarding the follow-up care plan [51]. Although none of the studies included direct analyses on the effects of cultural safety, three studies [51, 53, 54] evidenced increased patient satisfaction, decreases in ED presentations, hospital LOS and readmissions when adopting cultural safety related practices within their interventions. Application of cultural safety was evidenced through partnering with Aboriginal Community Controlled Health Services (ACCHs) [53], inclusion of cultural experts within multidisciplinary teams [51, 53, 54], ensuring patients where spoken to in their first language and use of local traditional values to connect with the patients [54].

Jayakody et al. (2018) reported a significant association between a 48-hour post-discharge telephone follow-up and reduction in unplanned ED presentations. The study conducted by Phillips et al. (2014) did not find statistically significant outcomes after using MMS [52]. The interventions and outcomes found are summarized with further detail in **Additional file 4**.

### Quality assessment

Four studies were classified as high quality [50–53] using the MMAT while the remaining study [54] was classified as medium quality by reviewers. The results of the quality assessment using the MMAT are reported and summarized **in Additional file 5.** Assessment of records using the CONSIDER statement checklist is available in **Additional file 6.** Only one study demonstrated high quality from the perspective of research involving First Nations people by reporting most items [51]. Another study reported 10 out of the 17 items [54] while the other studies reported less than half of the items. This made it difficult to assess whether the studies followed appropriate protocols when conducting research with First Nations people such as First Nations’ leadership and community engagement.

## Discussion

Our systematic review examined discharge interventions among First Nations people with chronic conditions to identify characteristics that improve continuity of care. This systematic review identified only one study conducted outside Australia and a limited number of Australian studies analysing effective discharge planning strategies for Aboriginal and Torres Strait Islander people. This highlights Australia’s leading research role in this space. It also supports the need for a research agenda that truly reflects the health needs and collaboration with First Nations communities [55, 56], which are best practice approaches recommended by NHMRC Ethical Guidelines[57, 58]. Programs implementing and evaluating interventions to improve continuity of care among First Nations people are clearly needed to achieve better health outcomes and inform broader effective public health initiatives to bridge the health gap [36, 37].

Ongoing racism continues to ensure a health gap in life is experienced by Aboriginal and Torres Strait Islander people in Australia [23, 59, 60]. The wide health disadvantage that First Nations peoples experience, compared to non-Indigenous people, has led to the evolution of First Nations models of care. These have been developed by and in partnership with First Nations representatives and aim to adequately meet the health needs of First Nations communities according to their cultural contexts and characteristics [61–64]. In 2018 Harfield performed a scoping review to identify the characteristics of First Nations primary health care service delivery models. The author found 8 main characteristics: accessible health services, community participation, continuous quality improvement, culturally appropriate and skilled workforce, culturally safe approach to care, holistic health care and self-determination [65].

Albeit limited by the small number of studies included, we observed that significant associations with positive health outcomes were found when implementing interventions which are in line with the main characteristics of First Nations managed primary health care service delivery model including First Nations health workers and trained professionals to coordinate follow-up appointments [65]. Patient training and education and links to local community services showed improvement in continuity of care and was reflected in lower hospital admissions, presentations to the emergency department, non-attended appointments and hospital length of stay.

The results support the importance of including First Nations health workforce to ensure continuity of care which is culturally safe [51, 53, 54]. First Nations Health Workers and Liaison Officers are cultural experts who work to support patients who have been admitted as an inpatient or outpatient in hospital settings. Aboriginal and Torres Strait Islander Health Workers and Liaison officer have similar roles in Australian hospital settings [66]. Aboriginal and Torres Strait Islander Health Workers provide important support services such as language translation and assistance with accessing food, transportation, accommodation and follow-up appointments [67]. These services enhance the circumstances to facilitate appropriate treatment adherence, and attendance to aftercare appointments. A study analysing the role of an Aboriginal Liaison Officer found that it serves as an initiator to facilitate access to services, a translator to assist with understanding among clinicians and patients and as a support worker and facilitator when discharging to the community providing cyclic continuity of care [68]. This suggests that ensuring effective aftercare for Aboriginal and Torres Strait Islander people requires having culturally experienced workers who can assist with communication issues between clinicians and patients and to organise culturally safe services that provide the necessary elements to facilitate culturally appropriate continuity of care.

Continuous health care is more likely to be effective when accessible health services link patients with heath care and social services that are culturally safe and available locally in their community [69–71]. Linking patients with local primary health professionals who have access to previous clinical history and can provide ambulatory or home health care has been recognised by the World Health Organization (WHO) as a priority to maintain continuity of care [72]. Another priority listed by the WHO is the case management of people with complex medical and social needs which includes integrating different community based services to meet those needs [72]. Other studies have also found that facilitating linkage and navigation between primary care and social services is necessary to meet patient’s health needs [73]. Thus, an effective discharge plan for Aboriginal and Torres Strait Islander people should include linkage and coordination with primary health and social services that are locally accessible for the patient.

The outcomes of the included studies indicate that interventions where patient and family education in medication use and ambulatory care training is provided are effective and important to help ensure optimal post discharge care. It has been recognised that patient education and health literacy when culturally appropriate are positively associated with population health and empowerment by facilitating informed autonomy over lifestyle choices and health related decision making [74, 75]. In 2018 The Mental Health Commission of NSW, in consultation with Aboriginal leaders, established community empowerment as one of the key criteria to the value and success of models of care for Aboriginal and Torres Strait Islander people [76]. Patient training and education promote health empowerment and self-determination. These are suggested as important elements for the success of discharge plans to achieve positive health outcomes.

The findings discussed above highlight that discharge interventions which adhere to the principal characteristics or domains of First Nations models of care can have positive impact in the health outcomes of Aboriginal and Torres Strait Islander people. The effectiveness of models of care which are led by Aboriginal and Torres Strait Islander people and truly consider Aboriginal and Torres Strait Islander worldviews and health understandings can be perfectly exemplified in Australia through the Aboriginal Community Controlled Health Organisations (ACCHOs). ACCHOs are Aboriginal community initiated and operated primary health care organisations that deliver holistic, comprehensive and culturally safe health care to the community that controls them [77]. ACCHOs have been crucial in addressing the negative health impacts of discrimination [65, 78] and have demonstrated high Aboriginal and Torres Strait Islander peoples engagement with satisfaction and positive outcomes in mental health, chronic conditions and antenatal care [79–82].

Another key component of First Nations primary health care service delivery model is culturally safe care. The studies that achieved increased patient satisfaction, reduction in emergency department presentations and hospital readmissions adopted cultural safety elements in their interventions [51, 53, 54]. Cultural safety in health care is fundamental to the positive health outcomes for First Nations people [83, 84]. Culturally safe healthcare involves dismantling disadvantages in health care faced by First Nations people, understanding culture, examining sources of repression, social domination, social justice, power imbalances and equity [26, 85]. Although cultural safety is widely recognised as a key element to improving First Nations people health [27, 86–88], current models of care in Australia do not address all aspects of cultural quality and safety [89]. It is thus understood that effective discharge interventions for Aboriginal and Torres Strait Islander people must embed principles of cultural safety in their healthcare delivery.

The use of communication technologies to implement discharge strategies showed mixed results. The use of post discharge phone follow-up was supported by the results of one study [90] which found a significant decrease of ED admissions while a study analysing the effect of MMS [52] did not find any significant impact. Synthesis research studying the effectiveness of these interventions among non-Indigenous population has found lack of high quality studies to demonstrate its benefit in hospital and primary care settings [91, 92]. Further studies are needed to assess the effectivity of strategies using communication technologies for post discharge interventions in the continuity of care in First Nations people.

### Strengths and limitations

Strengths of this study are the use of meticulous and transparent database search following standard guidelines, inclusion of multiple databases to conduct the search, the quality assessment of the included studies using a validated instrument and the use of a decolonising approach throughout the analysis of the results ensuring that principles of self-determination were respected [93, 94].

The most important limitation of this review was the low number of studies which met the inclusion criteria and their individual methodological quality. The scarce number of studies included in this review do not provide enough evidence to draw definitive conclusions. Furthermore, there is a high risk of publication bias since we did not exhaustively search for grey literature.

## Conclusion

Strategies are needed to increase access to ongoing care for Aboriginal and Torres Strait Islander people. The scarcity of published studies indicate that further research is needed to inform the design and implementation of programs to ensure access to ongoing care and improve health outcomes.

The findings of our review indicate that discharge interventions including First Nations workforce, coordination of accessible and culturally safe health services and patient education and empowerment, contribute to improving aftercare and are associated with positive health outcomes. These characteristics are in line with First Nations models of care which have been proven to be successful in providing culturally safe health services and achieving positive health outcomes. We suggest that discharge interventions for Aboriginal and Torres Strait Islander people should follow the domains of First Nations models of care.

## Electronic supplementary material

Below is the link to the electronic supplementary material.

Supplementary Material 1

Supplementary Material 2

Supplementary Material 3

Supplementary Material 4

Supplementary Material 5

Supplementary Material 6

## Data Availability

The datasets used and/or analysed during the current study are available from the corresponding author on reasonable request.

## List of Abbreviations

ACCHOS: Aboriginal Community Controlled Health Organisations
ACCHs: Aboriginal Community Controlled Health Services
ED: Emergency Department
LOS: Length of Stay
MMAT: Mixed Methods Appraisal Tool
MMS: Multimedia Messaging Service
PRISMA: Preferred Reporting Items for Systematic Review
WHO: World Health Organisation
