# Supplementary material for: Discharge interventions for First Nations people with a chronic condition or injury: a systematic review
Source: BMC Health Serv Res. 2023 Jun 9;23:604. doi: 10.1186/s12913-023-09567-5 (PMC10251590; doi:10.1186/s12913-023-09567-5)
Supplement: Supplementary file 2 — Supplementary Material 2 [file 12913_2023_9567_MOESM2_ESM.docx]

**Additional file 2. Search Strategy**

**PubMed :** 972 results

(((("indige*"[Title/Abstract] OR "aborigin*"[Title/Abstract] OR "Torres Strait Islander"[Title/Abstract] OR "First People"[Title/Abstract] OR "Inuit"[Title/Abstract] OR "First Nation"[Title/Abstract] OR "Maori"[Title/Abstract] OR "Native American"[Title/Abstract] OR "Native"[Title/Abstract] OR "Sami"[Title/Abstract]) AND "english"[Language]) OR ("Indigenous Peoples"[MeSH Terms] OR "Native Hawaiian or Other Pacific Islander"[MeSH Terms] OR "Indigenous Canadians"[MeSH Terms])) AND ("injur*"[Title/Abstract] OR "burn*"[Title/Abstract] OR "chronic condition*"[Title/Abstract] OR "chronic illness*"[Title/Abstract] OR "chronic disease*"[Title/Abstract] OR "Chronically ill"[Title/Abstract] OR "long term condition*"[Title/Abstract] OR "Chronic Disease"[MeSH Terms]) AND ("discharg*"[Title/Abstract] OR "Follow UP"[Title/Abstract] OR "Aftercare"[Title/Abstract] OR "Patient Discharge"[MeSH Terms]) AND ("english"[Language] AND 2000/01/01:2022/07/31[Date - Publication])) AND (english[Filter])

**CINAHL:** 468 results

S1 AND S2 ANS S3 (limiters: date published :01/01/2000-01/07/2022. Language: English) **(search mode: find all my search terms)**

S1: Indige* OR Aborigin* OR indian OR “Torres Strait Islander” OR “First People” OR Inuit OR “First Nation” OR Maori OR “Native American” OR Native OR Sami OR trib* **(search mode: Boolean/phrase)**

S2: Injur* OR Burns OR "Chronic condition" OR "Heart disease" OR “Chronic kidney disease” OR “Cystic fibrosis” OR Diabetes OR “COPD” OR "chronic obstructive pulmonary disease" OR "Chronic illness" OR "Chronic disease" OR "Chronically ill" OR "Long term condition"**(search mode: Boolean/phrase)**

S3: Discharge plan* OR discharge OR Patient discharge OR Discharge process OR Discharge management OR Discharge education OR Discharge intervention OR "Follow UP" OR Aftercare OR "Model of aftercare"**(search mode: Boolean/phrase)**

**Embase:** 1928 results

1. (indige* or aborigin* or "torres strait islander" or "first nation" or "first people" or "native american" or "native" or "maori" or "sami" or "inuit" or trib*).mp. [mp=title, abstract, heading word, drug trade name, original title, device manufacturer, drug manufacturer, device trade name, keyword, floating subheading word, candidate term word]
2. ("Discharge plan*" or discharge or "Patient discharge" or "Discharge process" or "Discharge management" or "Discharge education" or "Discharge intervention" or "Follow UP" or Aftercare).mp. [mp=title, abstract, heading word, drug trade name, original title, device manufacturer, drug manufacturer, device trade name, keyword, floating subheading word, candidate term word]
3. (injur* or burn* or "chronic disease" or "chronic condition" or "chronic illness" or "chronically ill" or "long term condition" or "long term disease").mp. [mp=title, abstract, heading word, drug trade name, original title, device manufacturer, drug manufacturer, device trade name, keyword, floating subheading word, candidate term word]
4. 1 and 2 and 3
5. limit 4 to (human and English language and yr="2000 - 2022")

**Web of Science:** 876 results

#1 AND #2 AND #3 and English (Languages)/ Timespan: 2000-01-01 to 2022-07-29 (Publication Date)

#1 : TS= (indige* OR aborigin* OR "torres strait islander" OR "first nation" OR "first people" OR "native american" OR "native" OR "maori" OR "sami" OR "inuit" OR Trib*)

#2: TS= (Injur* OR Burns OR "Chronic condition"OR "Chronic illness" OR "Chronic disease" OR "Chronically ill" OR "Long term condition")

#3: TS= ("Discharge plan*" or discharge or "Patient discharge" or "Discharge process" or "Discharge management" or "Discharge education" or "Discharge intervention" or "Follow UP" or Aftercare)

**ProQuest:** 260 results

noft(Indige* OR Aborigin* OR indian OR "Torres Strait Islander" OR "First People" OR Inuit OR "First Nation" OR Maori OR "Native American" OR Native OR Sami OR trib*) AND noft(injur* OR burn* OR "chronic disease" OR "chronic condition" OR "chronic illness" OR "chronically ill" OR "long term condition" OR "long term disease") AND noft("Discharge plan*" OR discharge OR "Patient discharge" OR "Discharge process" OR "Discharge management" OR "Discharge education" OR "Discharge intervention" OR "Follow UP" OR Aftercare)

Filters: English language, publication date ( 2000-01-21 to 2022-29-07), topic: Medicine
