# Supplementary material for: Discharge interventions for First Nations people with a chronic condition or injury: a systematic review
Source: BMC Health Serv Res. 2023 Jun 9;23:604. doi: 10.1186/s12913-023-09567-5 (PMC10251590; doi:10.1186/s12913-023-09567-5)
Supplement: Supplementary file 3 — Supplementary Material 3 [file 12913_2023_9567_MOESM3_ESM.docx]

**Additional file 3. Studies’ Characteristics.**

| **First author and year** | **Title** | **Country** | **Journal** | **Sample size** | **Sample source** | **Age range** | **Study Design** | **Objective** | **Research tool** |
| --- | --- | --- | --- | --- | --- | --- | --- | --- | --- |
| Cresp et.al (2016) | Effectiveness of the Koorliny Moort out-of-hospital health care program for Aboriginal and Torres Strait Islander children in Western Australia | Australia WA | Medical Journal of Australia | 942 | Referrals from Aboriginal Community controlled Health services, GP, allied health, Medicare, community workers and specialist Dr. | 0-16 years. | Experimental pre-test, post-test. | "To determine whether the Koorliny Moort program could  reduce emergency department presentations, hospital admissions and  length of stay, and improve attendance at out-of-hospital appointments for  Aboriginal and Torres Strait Islander children in Western Australia" | Occasions of service and person-time in days were compared for each child before and after referral to the program. |
| Jayakody, et al. (2018) | The impact of telephone follow-up on adverse events for Aboriginal people with chronic disease in new South Wales, Australia: a retrospective cohort study | Australia  NSW | International Journal for Equity in Health | 18659 | Linked data from the "48 hour follow up program" | >15 years | Retrospective cohort. | "To assess the impact of a telephone follow up program, 48 Hour Follow Up, on rates of unplanned hospital readmissions, unplanned emergency department presentations and mortality within 28 days of discharge among Aboriginal people with chronic disease." | Logistic regression models including confounding variables with 5% or more significant level. |
| Kim et al. (2019) | Ke Ku'una Na'au: A Native Hawaiian Behavioural Health initiative at The Queen's Medical Center | Hawaii | Hawaii Journal Med Public Health | 338 | Patients at the Queens Medical center screened at the bedside and deemed eligible for the program. | > 18 years | Experimental pre-test, post-test. | To describe the structure, history, and impact of the KKN initiative. | Comparison of readmission rates before and after program implementation. |
| Phillips et al. (2014) | Can mobile phone multimedia messages and text messages improve clinic attendance for Aboriginal children with chronic otitis media? A randomised controlled trial. | Australia  NT | Journal of Paediatrics and Child Health | 53 | Patients attending to the medical clinics with acute or chronic tympane membrane perforation. | < 13 years | Multi-centre, parallel group, randomised controlled trial. | To investigate the possible attendance and health benefits of sending regular MMS to families of children with tympanic membrane perforation. | Initial and 6-week end of intervention ear examinations using a tympanometer,a voroscope with Siegel’s speculum for pneumatic otoscopy and a video-otoscope. |
| Blignault et al. (2021) | “You Can’t Work with My People If You Don’t Know How to”: Enhancing Transfer of Care from Hospital to Primary Care for Aboriginal Australians with Chronic Disease | Australia NSW | Journal of Environmental Research and Public Health | 49 participants:  10 Aboriginal Transfer of Care team members,  20 hospital staff members,  9 Community-based service providers,  10 patients and their family/carers . | Patients and Aboriginal Transfer of Care stakeholders | Not specified | Qualitative | "To explore patient, family and service provider experiences and views and to document and refine the model of care for Aboriginal adults  with chronic conditions." | Semi structured interviews with patients and their families , staff members from the Aboriginal Transfer of Care model, hospital staff members and Primary care and community service providers. |
