# Supplementary material for: Discharge interventions for First Nations people with a chronic condition or injury: a systematic review
Source: BMC Health Serv Res. 2023 Jun 9;23:604. doi: 10.1186/s12913-023-09567-5 (PMC10251590; doi:10.1186/s12913-023-09567-5)
Supplement: Supplementary file 4 — Supplementary Material 4 [file 12913_2023_9567_MOESM4_ESM.docx]

**Additional file 4. Summary of interventions, outcomes and associations.**

| **Authors** | **Interventions** | **Studied outcomes** | **Outcomes and Significant Associations** |
| --- | --- | --- | --- |
| Cresp et al. (2016) | 1. partnership with primary care providers  2. Nurse-lead coordination for follow-up appointments.  3.Outreach care by paediatrician, nurse and social workers close to child's home.  4. Education, training and capacity building for ambulatory care. | - Emergency department presentations. - Hospital admissions. - Length of hospital stay. - Non-attended appointments | - Decrease in incidence of emergency presentations. - Decrease in hospital admissions. - Decrease in non-attended appointments. - Decrease in length of hospital stay. |
| Kim et al. (2019) | 1. Assignation to a community health worker. 2. Coordination of follow-up appointments. 3. Link to community resources. 4. Education on healthy lifestyle and medication use. | 30 days readmission rates. | - Reduction from 21% to 12% of patients in the KKN program in one year. - Reduction from 16.6% in 2015 to 12.6% in 2017 of annual readmission rates for Hawaiian patients. |
| Jayakody et al. (2018) | 48 hours telephone follow up | - Unplanned hospital readmissions within 28 days of discharge from acute facility. - Mortality within 28 days of discharge. - Unplanned ED presentation within 28 days of discharge. | - Reduction in unplanned ED presentation. |
| Phillips et al. (2014) | Seven ear health MMS in local Indigenous language (one every 4 days) about importance of hearing in Aboriginal context and a prompt to visit the clinic for health check-ups. | - Number of clinic appointments attended for any reason. - Ear health state at the end of study (healed tympanic membrane, ear discharge, perforation size). - Participant satisfaction with MMS. | No statistically significant differences. |
| Blignault et al (2021) | The Aboriginal Transfer of Care (ATOC) model had five key elements:  1. Transfer of care planning by a multidisciplinary team.  2. Ensuring the patient and their family understand the follow-up care plan.  3. Ensuring the patient’s General Practitioner (GP) or Aboriginal Medical Service (AMS)  is aware of any follow-up arrangements.  4. Ensuring referrals are organised with community providers.  5. Ensuring the patient has the necessary medications, equipment and written patient  summary information prior to transfer of care. | - patients' experience being prepared for and leaving the hospital. - ATOC team members and relevant clinicians' views on the model of care, its strengths, weaknesses, barriers and enablers to its implementation and sustainability. - Community service providers' views on continuity and coordination of care of patients after ATOC implementation. | - Integration of the 5 elements of ATOC contributes to effective transfer and positive patient journey by: 1 ) A multidisciplinary team bringing clinical and cultural expertise to meet individual's needs and preferences, 2)Ensuring effective communication between providers and family, 3) Ensuring access to other required services such as home care services and 4) Ensuring medication, home set up and equipment and follow up visits are organised previous to hospital discharge. - Outcomes: decrease in unplanned readmissions and ED presentations, increase in Aboriginal patient identification, improved patient experience , stronger partnership reported between services. - Challenges: staff availability, ATOC operating only from 9am to 5pm, inadequate identification of Aboriginal patients , broader culturally unsafe services across the hospital. - Success factors: strong governance with clinician and cultural leadership, multidisciplinary team working collaboratively, Availability of male and female ALOs to respect cultural protocols. |
