# Supplementary material for: Discharge interventions for First Nations people with a chronic condition or injury: a systematic review
Source: BMC Health Serv Res. 2023 Jun 9;23:604. doi: 10.1186/s12913-023-09567-5 (PMC10251590; doi:10.1186/s12913-023-09567-5)
Supplement: Supplementary file 5 — Supplementary Material 5 [file 12913_2023_9567_MOESM5_ESM.docx]

**Additional file 5. Quality assessment of the studies using the Mixed Methods Appraisal Tool (MMAT).**

| **Authors** | **Title** | **Study category** | **Reviewer 1** | **Reviewer 2** |
| --- | --- | --- | --- | --- |
| Cresp, et al. (2016) | Effectiveness of the Koorliny Moort out-of-hospital health care program for Aboriginal and Torres Strait Islander children in Western Australia. | Quantitative non-randomized | 5 | 5 |
| Jayakody, et al. (2018) | The impact of telephone follow-up on adverse events for Aboriginal people with chronic disease in new South Wales, Australia: a retrospective cohort study. | Quantitative non-randomized | 5 | 5 |
| Kim, et al. (2018) | Ke Ku'una Na'au: A Native Hawaiian Behavioural Health initiative at The Queen's Medical Center. | Quantitative non-randomized | 3 | 3 |
| Phillips, et al. (2014) | Can mobile phone multimedia messages and text messages improve clinic attendance for Aboriginal children with chronic otitis media? A randomised controlled trial. | Quantitative randomized controlled trials | 5 | 5 |
| Blignault, et al. (2021) | “You Can’t Work with My People If You Don’t Know How to”:  Enhancing Transfer of Care from Hospital to Primary Care for  Aboriginal Australians with Chronic Disease | Qualitative | 5 | 5 |

- **Low quality:** 0-1
- **Medium quality:** 2-3
- **High quality:** 4-5
