# Supplementary material for: Discharge interventions for First Nations people with a chronic condition or injury: a systematic review
Source: BMC Health Serv Res. 2023 Jun 9;23:604. doi: 10.1186/s12913-023-09567-5 (PMC10251590; doi:10.1186/s12913-023-09567-5)
Supplement: Supplementary file 6 — Supplementary Material 6 [file 12913_2023_9567_MOESM6_ESM.docx]

**Additional file 6. Quality appraisal using the Consolidated criteria for strengthening the reporting of health research involving Indigenous Peoples (CONSIDER) statement.**

| **Checklist item** | **Cresp, et al. (2016)** | **Jayakody, et al. (2018)** | **Kim, et al. (2018)** | **Phillips, et al. (2014)** | **Blignault, et al. (2021)** |
| --- | --- | --- | --- | --- | --- |
| **Governance** | | | | | |
| 1. Describe partnership agreements between the research institution and Indigenous-governing organization for the research, (e.g., Informal agreements through to MOU (Memorandum of Understanding) or MOA (Memorandum of Agreement)) | No | No | Yes | No | Yes |
| 2. Describe accountability and review mechanisms within the partnership agreement that addresses harm minimization. | No | No | No | No | Yes |
| 3. Specify how the research partnership agreement includes protection of Indigenous intellectual property and knowledge arising from the research, including financial and intellectual benefits generated (e.g., development of traditional medicines for commercial purposes or supporting the Indigenous community to develop commercialization proposals generated from the research). | No | No | No | No | Yes |
| **Priorization** | | | | | |
| 4. Explain how the research aims emerged from priorities identified by either Indigenous stakeholder, governing bodies, funders, non-government organization(s), stakeholders, consumers, and empirical evidence | Partially | Partially | Yes | Partially | Yes |
| **Relationships** | | | | | |
| 5. Specify measures that adhere and honour Indigenous ethical guidelines, processes, and approvals for all relevant Indigenous stakeholders, recognizing that multiple Indigenous partners may be involved, e.g., Indigenous ethics committee approval, regional/national ethics approval processes. | Partially | Partially | Yes | Partially | Yes |
| 6. Report how Indigenous stakeholders were involved in the research processes (i.e., research design, funding, implementation, analysis, dissemination/recruitment) | Yes | No | Yes | Partially | Yes |
| 7. Describe the expertise of the research team in Indigenous health and research. | Partially | No | No | No | Partially |
| **Methodologies** | | | | | |
| 8. Describe the methodological approach of the research including a rationale of methods used and implication for Indigenous stakeholders, e.g., privacy and confidentiality (individual and collective) | Partially | Partially | Partially | Partially | Yes |
| 9. Describe how the research methodology incorporated consideration of the physical, social, economic and cultural environment of the participants and prospective participants. (e.g., impacts of colonization, racism, and social justice). As well as Indigenous worldviews. | No | No | Yes | No | Yes |
| **Participation** | | | | | |
| 10. Specify how individual and collective consent was sought to conduct future analysis on collected samples and data (e.g., additional secondary analyses; third parties accessing samples (genetic, tissue, blood) for further analyses). | No | No | No | No | No |
| 11. Described how the resource demands (current and future) placed on Indigenous participants and communities involved in the research were identified and agreed upon including any resourcing for participation, knowledge, and expertise | No | No | No | No | Yes |
| 12. Specify how biological tissue and other samples including data were stored, explaining the processes of removal from traditional lands, if done, and of disposal. | No | No | No | No | Partially |
| **Capacity** | | | | | |
| 13. Explain how the research supported the development and maintenance of Indigenous research capacity (e.g., specific funding of Indigenous researchers). | No | No | Partially | No | Yes |
| 14. Discuss how the research team undertook professional development opportunities to develop the capacity to partner with Indigenous stakeholders? | No | No | Partially | No | Yes |
| **Analysis and interpretation** | | | | | |
| 15. Specify how the research analysis and reporting supported critical inquiry and a strength-based approach that was inclusive of Indigenous values. | No | No | Partially | No | Yes |
| **Dissemination** | | | | | |
| 16. Describe the dissemination of the research findings to relevant Indigenous governing bodies and peoples. | No | No | No | No | Yes |
| 17. Discuss the process for knowledge translation and implementation to support Indigenous advancement (e.g., research capacity, policy, investment). | No | No | Partially | No | Yes |

**Yes:** There was explicit and clear information indicating that the item was addresses during research design or conduct.
**Partially:** The article had some information indicating that the item was addressed.

**No:** The article did not provide information indicating weather or not the item was addressed.
